# Phase Transition Behaviors of Poly(*N*-isopropylacrylamide) Nanogels with Different Compositions Induced by (−)-Epigallocatechin-3-gallate and Ethyl Gallate

**DOI:** 10.3390/molecules28237823

**Published:** 2023-11-28

**Authors:** Ke Deng, Yafei Wang, Lei Wang, Xianli Fan, Zhenyu Wu, Xue Wen, Wen Xie, Hong Wang, Zheng Zhou, Pengfei Chen, Xianggui Chen

**Affiliations:** 1 School of Food and Bioengineering, Xihua University, Chengdu 610039, China; kelvin_de@126.com (K.D.); wyf@stu.xhu.edu.cn (Y.W.); 212021086000031@stu.xhu.edu.cn (L.W.); 18383013920@139.com (X.F.); zhenyuwu1807@gmail.com (Z.W.); zhouzheng@mail.xhu.edu.cn (Z.Z.); 1220180037@mail.xhu.edu.cn (P.C.); 2 School of Life Science and Engineering, Southwest Jiaotong University, Chengdu 610031, China; wenxue0703@my.swjtu.edu.cn (X.W.); xiewen@my.swjtu.edu.cn (W.X.);

**Keywords:** thermo-responsive nanogel, poly(*N*-isopropylacrylamide-*co*-*N*-hydroxymethyl acrylamide), poly(*N*-isopropylacrylamide-*co*-2-hydroxyethyl methacrylate), (−)-epigallocatechin-3-gallate, phase transition

## Abstract

Phase transition behaviors of poly(*N*-isopropylacrylamide) nanogels with different compositions induced by (−)-epigallocatechin-3-gallate (EGCG) and ethyl gallate (EG) has been investigated systematically. Monodisperse poly(*N*-isopropylacrylamide-*co*-*N*-hydroxymethyl acrylamide) (P(NIPAM-*co*-NMAM)) and poly(*N*-isopropylacrylamide-*co*-2-hydroxyethyl methacrylate) (P(NIPAM-*co*-HEMA)) nanogels with different feeding monomer ratios were prepared by emulsion polymerization. P(NIPAM-*co*-NMAM) nanogels exhibit rapid isothermal phase transition behavior in EGCG solutions with low concentration (10^−3^ mol/L) in less than 10 minutes. The thermosensitive phase transition behaviors of nanogels are affected not only by the copolymerized monomers but also by the concentrations of EGCG and EG in aqueous solutions. Nanogels remain in a shrunken state and do not exhibit thermosensitive phase transition behaviors in EGCG solutions (≥5 mmol/L), whereas they display thermo-responsive phase transition behaviors in EG solutions. The volume phase transition temperature (VPTT) shifts to lower temperatures with increasing EG concentration. The diameters of P(NIPAM-*co*-NMAM) nanogels decrease with increasing EG concentration at temperatures between 29 and 33 °C. In contrast, the diameters of P(NIPAM-*co*-HEMA) nanogels increase with increasing EGCG concentration at temperatures between 37 and 45 °C. The results demonstrate the potential of nanogels for simple detection of EG and EGCG concentrations in aqueous solutions over a wide temperature range, and EGCG can serve as a signal for the burst-release of drugs from the P(NIPAM-*co*-NMAM)-based carriers at physiological temperature.

## 1. Introduction

Polyphenols, as widely existing natural bioactive compounds including simple phenols with low molecular weight and highly polymerized polyphenols, provide broad and advanced applications in the fields of food, medicine, biology, and chemistry due to their versatile biofunctions such as anti-inflammatory [1], immune-regulating [2], antibacterial [3], and antioxidant activities [4,5]. Polyphenols are crucial for functions like cyto-protection, pigmentation, metal coordination, iron transport, and signal transduction [6]. In the food industry, polyphenols primarily serve as natural food preservatives, improving food storage quality, extending shelf life, and preserving the color and flavor of food [7]. Polyphenols can also be used as carriers for drug delivery or as active ingredients for anticancer, antibacterial, and free-radical scavenging [8,9,10]. Polyphenol-based biomaterials can promote tissue and bone regeneration as wound dressings or scaffolds [11]. The polyphenol compounds could dynamically interact and bind with diverse species, such as metal ions, proteins, polysaccharides, and polymers [12,13]. Understanding of interactions between polyphenols and these species holds great significance in food processing, nutritional digestion, drug delivery, sensing, and detection [14,15].

Thermo-responsive polymers were widely used in sensing and drug delivery due to the facile tunability of temperature [16,17]. Compared with other thermosensitive polymers, poly(*N*-isopropylacrylamide) (PNIPAM) hydrogel is easy to polymerize and tough in sensitive environments, with an attractive lower critical solution temperature (LCST) around 32 °C [17], which is between room and body temperature. Therefore, PNIPAM is ideal for developing thermo-responsive hydrogels for biomedical applications [18]. It exhibits hydrophilic properties, with polymer chains swelling in water below the LCST. When the temperature exceeds the LCST, the hydrogen bonds between the amide groups of PNIPAM and water molecules are broken, causing the polymer chains to shrink due to strong hydrophobic interactions among the isopropyl groups in the networks. The benzene rings and hydroxyl groups in polyphenols can form robust hydrophobic and hydrophilic interactions with the isopropyl and amide groups in PNIPAM [19]; thus, the presence of polyphenol molecules in solutions could change the volume phase transition of PNIPAM, altering its LCST [20,21]. László et al. studied the host–guest interactions in PNIPAM hydrogels and found that small aromatic molecules dissolved in PNIPAM hydrogels modify the LCST by partially shielding the NIPAM side-chains from the solvent molecules and disrupting their organization [22,23]. Koga et al. investigated how benzoic acid and phenol molecules influence the phase transition behavior of PNIPAM. They observed that the LCST of PNIPAM shifted to lower temperatures as a result of the adsorption of benzoic acid or phenol molecules onto the polymer chains [19]. Chu et al. used crosslinked PNIPAM microspheres rather than linear PNIPAM chains to study the effect of tannic acid on the phase transition behavior of PNIPAM and found that the thermo-responsive phase transition of PNIPAM microgels is dependent on the tannic acid concentration [24]. Chu et al. also showed that the volume phase transition temperature (VPTT) of PNIPAM core-shell microcapsules in an aqueous solution of ethyl gallate (EG) decreases as the EG concentration increases [25]. These investigations demonstrated that PNIPAM shows high potential for use as sensors to detect polyphenol concentration and as stimulation signals for releasing drugs from PNIPAM-based vehicles. However, pure PNIPAM microspheres or macroscale hydrogels interacting with polyphenols result in long equilibration times and a narrow operating temperature range (below 32 °C), which limits the application of polyphenol–PNIPAM interactions in the food industry and drug delivery.

Nanogels, with a larger specific surface area and faster response rate, are widely used in the food and pharmaceutical industries [26,27]. For example, poly(*N*-isopropylacrylamide-*co*-acrylic acid) nanogel as a doxorubicin (DOX) carrier can be phagocytized by 4T1 tumor cells and release DOX in tumor cell acidic environments [28], which is promising for synergetic chemo-photothermal therapy of tumors. The phase transition temperature of PNIPAM hydrogel could be adjusted by the copolymerized monomers. By copolymerizing hydrophilic monomer *N*-hydroxymethyl acrylamide (NMAM) [26] with NIPAM at a certain ratio, the VPTT of PNIPAM hydrogel may increase to close to physiological temperature [29]. The introduction of 2-hydroxyethyl methacrylate (HEMA) into the PNIPAM network may lower the VPTT of the hydrogel [30]. 3,4,5-trihydroxybenzoic acid ethyl ester (EG) is a typical gallic acid alkane ester with three phenol hydroxyl groups and is abundant in medicinal plants and fermented beverages [31]. (−)-Epigallocatechin-3-gallate (EGCG) is the most potent active component of tea polyphenols [32], containing more phenol hydroxyl groups compared with EG [33]. However, despite EGCG being a crucial substance in the food and pharmaceutical industry, its effect on the volume phase transition of PNIPAM-based thermo-responsive hydrogel has not been reported yet. Therefore, studying the phase transition behavior of nanogels triggered by EG and EGCG over a wide temperature range is important for understanding the interactions between thermo-responsive nanogels and polyphenol and for developing drug delivery vehicles with burst-release properties at physiological temperature [25].

In this study, to achieve the rapid phase transition behaviors of nanogels induced by EG and EGCG with a wide range of temperature, we prepared thermosensitive nanogels with different compositions by copolymerizing NIPAM with NMAM or HEMA at a certain molar ratio. Then the phase transition behavior of P(NIPAM-*co*-NMAM) and P(NIPAM-*co*-HEMA) nanogels in response to different concentrations of EG and EGCG at temperatures ranging from 5 to 45 °C was investigated. In detail, the effect of different monomer ratios on the thermo-responsive phase transition behaviors of nanogels was firstly studied. Then, the isothermal volume changes of nanogels in response to EG and EGCG solutions with varied concentrations were investigated. Finally, the response behaviors of P(NIPAM-*co*-NMAM) and P(NIPAM-*co*-HEMA) nanogels to different concentrations of EG and EGCG at different temperatures were systematically examined. The results in this study provide new insights into the interactions between polyphenol molecules and PNIPAM nanogels with different compositions.

## 2. Results and Discussion

### 2.1. Synthesis and Characterization of Nanogels

**Figure 1:**
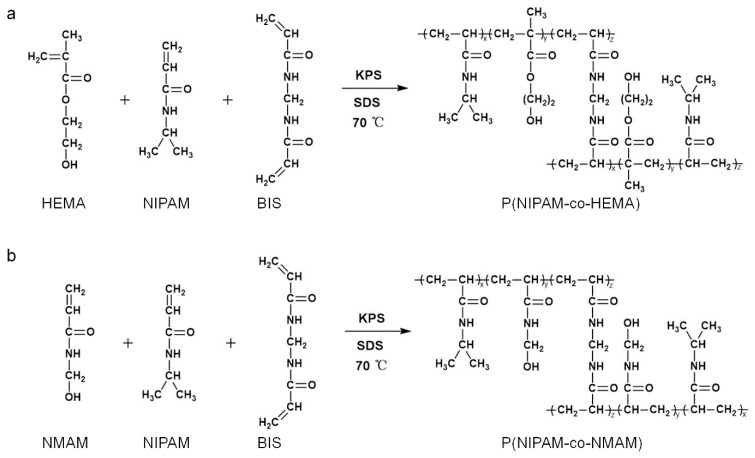
Synthesis of P(NPAM-*co*-HEMA) (**a**) and P(NIPAM-*co*-NMAM) (**b**) nanogels.

**Figure 2:**
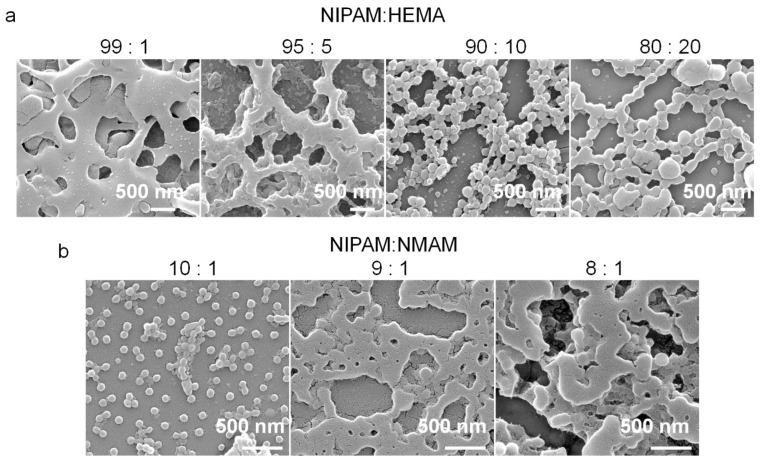
SEM images of P(NPAM-*co*-HEMA) (**a**) and P(NIPAM-*co*-NMAM) (**b**) nanogels with different monomer molar ratios in the dry state.

**Figure 3:**
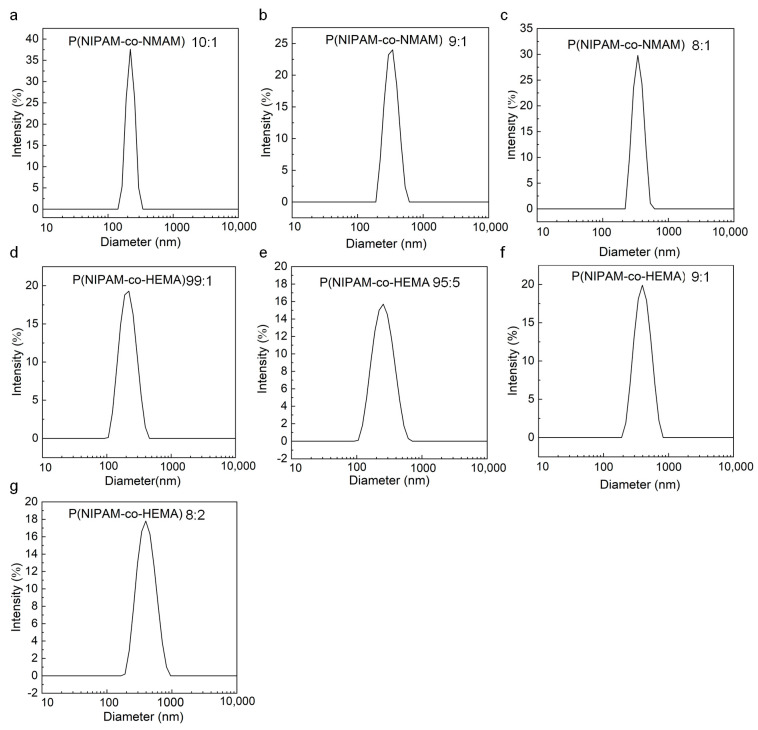
Good monodespersity of nanogels. (**a**–**c**) Diameter distribution of P(NIPAM-*co*-NMAM) with different monomer ratios of 10:1 (**a**), 9:1 (**b**), and 8:1 (**c**). (**d**–**g**) Diameter distribution of P(NIPAM-*co*-HEMA) with different monomer ratios of 99:1 (**d**), 95:5 (**e**), 9:1 (**f**), and 8:2 (**g**).

The nanogels with thermo-responsive properties were synthesized through emulsion polymerization [30] (Figure 1). PNIPAM microgels are often prepared via precipitation polymerization. However, this method results in P(NPAM-*co*-HEMA) and P(NPAM-*co*-NMAM) gels with poor monodispersity and submicron size. Here, sodium dodecyl sulfate (SDS) was added to the reaction system to ensure the nano-size of gel diameter with narrow size distribution. SEM images depict the morphology of the nanogels, displaying a spherical shape in the dry state (Figure 2). Due to the hydrophobic interaction between nanogels during the drying process, some nanogels, especially P(NIPAM-*co*-HEMA), aggregated after water evaporated, as shown in Figure 2. It should be noted that the nanogels are well-dispersed in aqueous solution, exhibiting good monodispersity without aggregation (Figure 3, Table S1). The diameters of nanogels containing NMAM or HEMA in the dry state range from 80 to 200 nm and increase with higher content of NMAM or HEMA. When the nanogels are swollen in water at 25 °C, the hydrodynamic diameters of P(NPAM-*co*-HEMA) nanogels with NIPAM:HEMA ratios of 99:1, 95:5, 90:10, and 80:20 are about 170 nm, 180 nm, 330 nm, and 350 nm, respectively. The hydrodynamic diameters of P(NPAM-*co*-NMAM) nanogels with NIPAM:NMAM ratios of 10:1, 9:1, and 8:1 are approximately 320 nm, 340 nm, and 360 nm in water, respectively.

**Figure 4:**
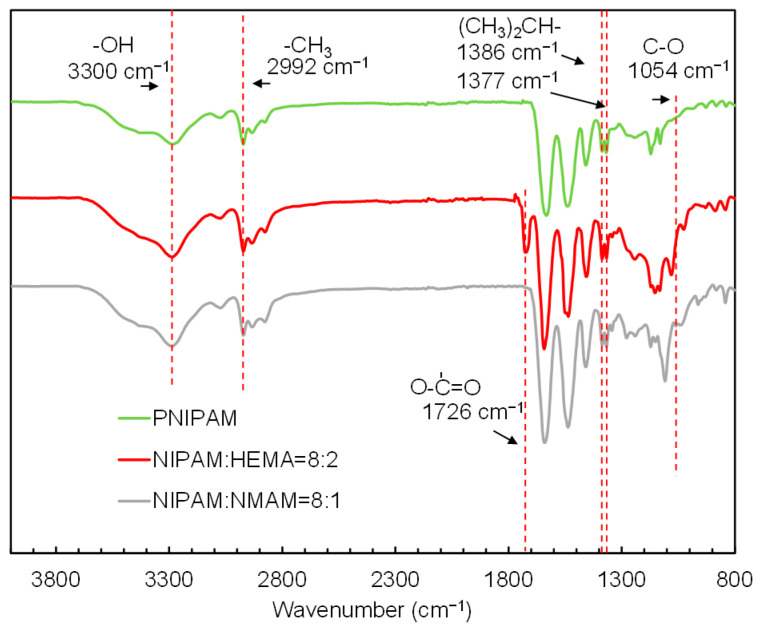
FT-IR spectra of PNIPAM microgels, P(NPAM-*co*-HEMA) nanogels, and P(NIPAM-*co*-NMAM) nanogels.

Further, the FT-IR spectra analysis was employed to identify the functional groups of the nanogels (Figure 4). The peaks present around 3300 cm^−1^ and 2992 cm^−1^ are due to the stretching frequency of -OH and -CH_3_ groups, respectively. The spectrum of the PNIPAM exhibits characteristic peaks of C-C vibrations of isopropyl groups at 1386 and 1377 cm^−1^ [34]. The peak around 1726 cm^−1^ is assigned to the carbonyl group of HEMA [35], indicating that the HEMA was copolymerized with NIPAM successfully. Additionally, a peak at 1054 cm^−1^ is observed for C-O stretching vibrations [36], confirming the successful synthesis of P(NIPAM-*co*-NMAM).

The thermal stability and degradation of the synthesized PNIPAM microgel and P(NIPAM-*co*-NMAM) nanogels with different NIPAM:NMAM ratios were studied using thermogravimetric analysis (Figure S1). The weight loss before 100 °C can be attributed to the loss of water molecules that are hydrogen-bonded to the hydrophilic amide groups of the polymer network. Above 100 °C in the DTG curve, PNIPAM microgel exhibits a single obvious peak around 410 °C, indicating a singular degradation mechanism of NIPAM content, whereas the nanogel exhibits two or three peaks, the shapes and positions of which are dependent on composition. The total degradation of PNIPAM is observed in two stages and is completed by about 420 and 550 °C, attributed to the decomposition of monomer NIPAM and crosslinker MBA [37]. As the content of NMAM increases, the temperature of total degradation of the nanogels also increases, indicating greater thermal stability of the nanogel. Compared with PNIPAM microgels, the P(NIPAM-*co*-NMAM) nanogel with a monomer feeding ratio of 10:1 undergoes weight losses of 56.47% near 400 °C and 30.89% near 600 °C, caused by the decomposition of NIPAM and NMAM monomers. The steady decrease in weight loss by about 60% of over the heating process (Figure S1d) indicates that the increasing NMAM content leads to an increase in thermal stability, which is attributed to the interaction between NIPAM segments and NMAM segments [38].

### 2.2. Thermo-Responsive Properties of Nanogels with Different Compositions

**Figure 5:**
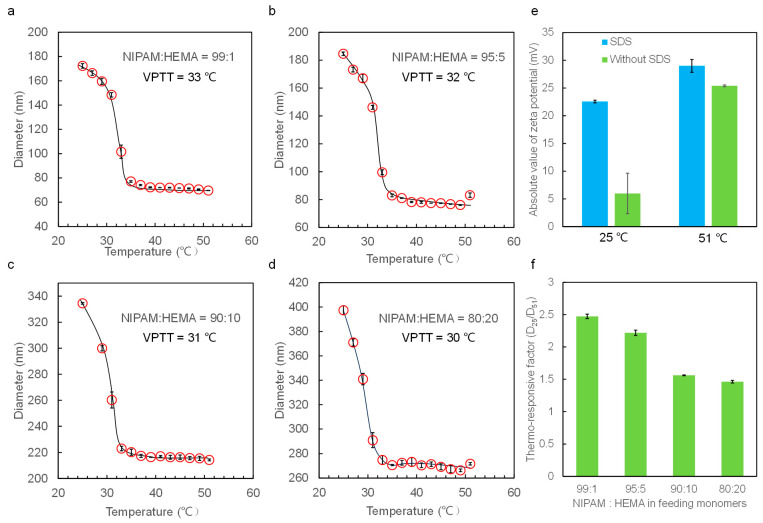
(**a**–**d**) Thermo-responsive hydrodynamic diameters of P(NIPAM-*co*-HEMA) nanogels with different compositions in water. (**e**) Absolute value of zeta potential of P(NIPAM-*co*-HEMA) nanogels with monomer ratio of 8:2 prepared with and without SDS. (**f**) Thermo-responsive factor of nanogels with different feeding monomers.

**Figure 6:**
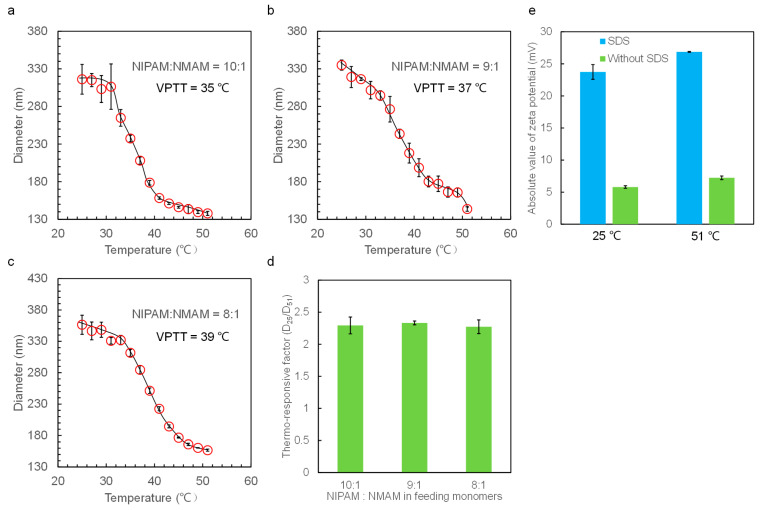
(**a**–**c**) Thermo-responsive hydrodynamic diameters of P(NIPAM-*co*-NMAM) nanogels with different compositions in water. (**d**) Thermo-responsive factor of nanogels with different feeding monomer ratios. (**e**) Absolute value of zeta potential of P(NIPAM-*co*-NMAM) nanogels with monomer ratio of 8:1 prepared with and without SDS.

DLS was employed to measure the hydrodynamic diameters of the nanogels in DI water at temperatures ranging from 25 to 51 °C. The diameters of the nanogels obtained from DLS are average values derived from a large number of nanogels, ensuring reliability in statistics. The zeta potential value of the nanogels is about −25 mV when adding SDS in the polymerization process, whereas the zeta potential is only about −5 mV without adding SDS (Figure 5e and Figure 6e). The larger zeta potential absolute value of the nanogels hinders their aggregation, endowing them with better monodispersity (Figure 3 and Figure S2, Table S1). Therefore, all the nanogels were prepared by adding SDS to the reaction solution.

The thermo-responsive phase transition behaviors of nanogels in aqueous solutions are observed. The nanogels are at swelling equilibrium in the pure water at the tested temperature before diameter measurement. DLS data show that all the nanogels exhibit good thermo-responsive volume changes in water around the VPTT, similarly to the previous work [39]. We determined the VPTT from the curve of nanogel diameters with temperatures, and the point where the diameters changed dramatically represents the VPTT value. The VPTT value of P(NIPAM-*co*-HEMA) nanogels decreases with an increase in HEMA content and is around 33 °C (NIPAM:HEMA = 99:1), 32 °C (NIPAM:HEMA = 95:5), 31 °C (NIPAM:HEMA = 90:10), and 30 °C (NIPAM:HEMA = 80:20), respectively. However, when introducing the hydrophilic monomers to the PNIPAM network, the VPTT of P(NIPAM-co-NMAM) nanogels increases and is 35 °C (NIPAM:NMAM = 10:1), 37 °C (NIPAM:NMAM = 9:1), and 39 °C (NIPAM:NMAM = 8:1). 

With the hydrophobic HEMA monomers replaced by the relatively hydrophilic NMAM, the diameters of nanogels relatively decrease with increasing temperatures above the VPTT (Figure 6). This continuous decrease in nanogel diameter above VPTT is due to the cleavage of hydrogen bonds between NMAM and water molecules. The thermo-responsive factors of the P(NIPAM-*co*-HEMA) decrease from about 2.5 to 1.5 with increasing HEMA content, whereas the thermo-responsive factors of P(NIPAM-*co*-NMAM) remain constant at around 2.3 with all the studied monomer ratios. These thermo-responsive factors represent the diameter change of nanogels with temperature variation and are defined by calculating the diameter of nanogels at 25 °C (which is below VPTT) divided by that at 51 °C (which is higher than VPTT). 

**Figure 7:**
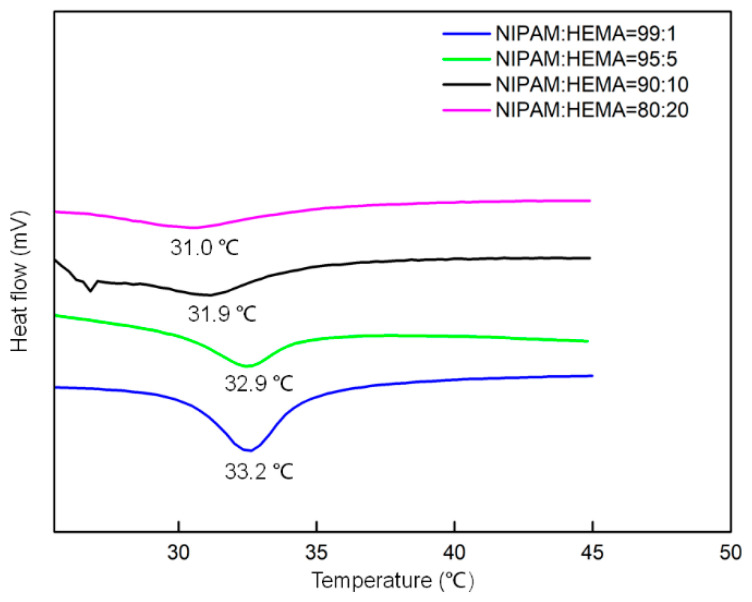
DSC response under heating of powdered P(NIPAM-*co*-HEMA) nanogels in DI water. Successive curves are shifted vertically.

In addition to being measured by the light-scattering method, the VPTT can also be obtained using the DSC method. DSC allows the measurement of the heat in phase transition of thermo-responsive polymers over a wide range of temperatures. Figure 7 illustrates the typical changes in the thermograms with increasing HEMA content. The results are consistent with the VPTT obtained from DLS method. The LCST shifts toward lower values, the endothermic peak becomes much narrower with increasing the HEMA content in nanogels, and the heat uptake is less steep as the onset temperature is reached.

### 2.3. Dynamic Isothermal Volume Change of Nanogels Induced by EG and EGCG

The good monodispersity of nanogels in EG and EGCG solutions is important for studying phase transition behavior of nanogels using the DLS method. We found that nanogel solution exposed to EG shows uniform properties without precipitate or phase separation of the solution at both 25 and 45 °C (Figure S2, Table S2). The uniformly dispersed nanogels in polyphenol solution ensure the accuracy of diameter measurement by DLS.

**Figure 8:**
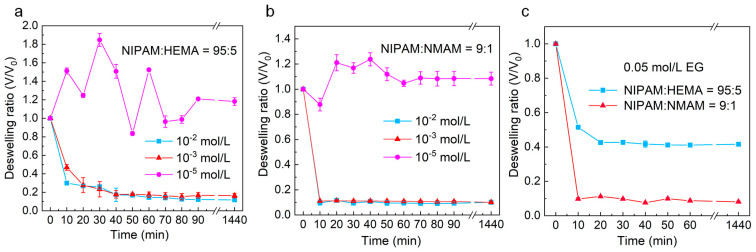
Dynamic volume change (V/V_0_) of P(NIPAM-*co*-HEMA) (**a**) and P(NIPAM-*co*-NMAM) (**b**) nanogels in EGCG solutions with different concentrations (**a**,**b**) and EG solution (**c**) at 25 °C.

Figure 8 illustrates the dynamic isothermal volume change behaviors of nanogels in solutions with different EG or EGCG concentrations at 25 °C. The results demonstrate that the volumes of nanogels do not change much at lower EGCG concentrations (10^−5^ mol/L). Interestingly, the volumes of nanogels slightly increase when immersed in lower EGCG concentrations at 25 °C due to the binding of more water molecules by EGCG in the polymer network. As the EGCG concentration increases to 10^−3^ and 10^−2^ mol/L, the nanogels exhibit a significant isothermal volume change. The higher the EGCG concentration, the faster the isothermal volume change of nanogels induced by EGCG. We also observed that the P(NIPAM-*co*-NMAM) nanogels respond to the polyphenol molecules more rapidly and undergo a larger volume change than P(NIPAM-*co*-HEMA) nanogels. The equilibrium time for P(NIPAM-*co*-HEMA) nanogels to reach the completely shrinking state in 10^−3^ mol/L EGCG solutions is about 50 min, whereas the equilibrium time for P(NIPAM-*co*-NMAM) nanogels in EG or EGCG solution is less than 10 min. The difference in equilibrium time is attributed to the faster diffusion rate of EGCG and EG in the P(NIPAM-*co*-NMAM) hydrogel network and the stronger binding capacity of P(NIPAM-*co*-NMAM) hydrogel with EGCG and EG.

### 2.4. Thermo-Responsive Phase Transition Behaviors of Nanogels at Different Concentrations of EG and EGCG

**Figure 9:**
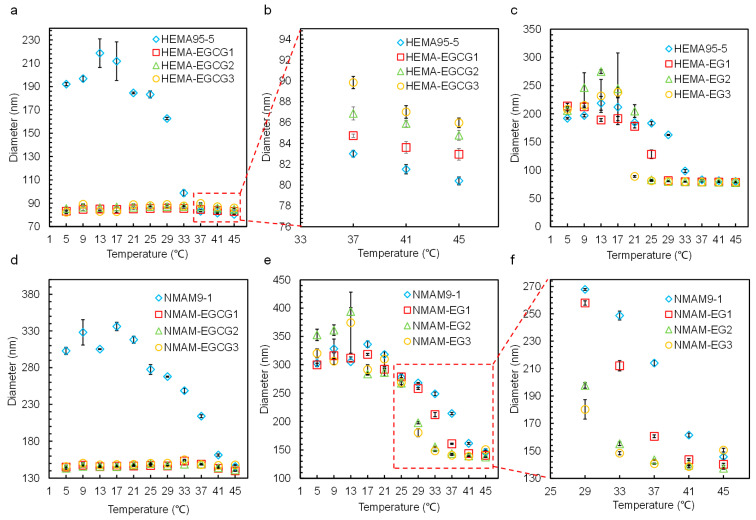
Thermo-responsive hydrodynamic diameters of P(NIPAM-*co*-HEMA) (**a**–**c**) and P(NIPAM-*co*-NMAM) (**d**–**f**) nanogels in EG (**c**,**e**,**f**) and EGCG (**a**,**b**,**d**) aqueous solutions with 0, 5, 10, and 15 mmol/L concentration. HEMA95-5 and NMAM9-1 represent the NIPAM:HEMA = 95:5 and NIPAM:NMAM = 9:1, respectively. EGCG1, EGCG2, and EGCG3 indicate that the concentrations of EGCG are 5, 10, and 15 mmol/L. EG1, EG2, and EG3 indicate that the concentrations of EG are 5, 10, and 15 mmol/L.

Figure 9 illustrates the thermo-responsive phase transition behaviors of nanogels in equilibrium with EG and EGCG solutions with different concentrations. In EGCG solutions, the nanogels shrink to a smaller size and do not exhibit significant thermo-responsive phase transition behavior. However, in EG solution with varied concentrations, the nanogels exhibit thermo-responsive behaviors. It has been reported that hydrogen bonds can form between the hydroxyl groups of phenols and the amide groups of PNIPAM in aqueous solutions [40]. Polyphenols like EGCG and EG molecules can interact with PNIPAM polymeric chains via hydrogen bonding and hydrophobic interactions. EGCG molecules, with more phenolic hydroxyl groups and benzene rings compared with EG, can interact more strongly with the P(NIPAM-*co*-HEMA) and P(NIPAM-*co*-NMAM) network, providing more binding sites through hydrophilic and hydrophobic interactions. This allows EGCG to act as physical crosslinker for the hydrogel networks [24]. The EGCG molecules crosslink the polymeric networks, displacing the water molecules initially situated at the adsorption site and reducing the distance between polymer chains. As a result, when enough EGCG molecules are bound to the polymeric networks, the nanogels shrink at all the tested temperatures. Additionally, the aromatic groups of EGCG molecules enhance the hydrophobic interaction of polymeric networks, disrupting the original equilibrium between hydrophilic and hydrophobic interactions. This also leads to the shrinkage of nanogels. 

As shown in Figure 9a,d, the diameters of nanogels in DI water are larger than those in EGCG solutions over a wide temperature range. As the temperature increases to a certain extent, the diameter of nanogels in DI water decreases and becomes no larger than the diameter of nanogels in EGCG solutions. We defined the temperature at which the diameters of nanogels in DI water is no larger than those in EGCG solutions as the critical temperature (*T*_c_). *T*_c_ is determined by the composition of hydrogel. For P(NIPAM-*co*-HEMA) nanogels with NIPAM:HEMA = 95:5, *T*_c_ is 37 °C, and for P(NIPAM-*co*-NMAM) nanogels with NIPAM:NMAM = 9:1, *T*_c_ is 45 °C. Below the *T*_c_, we can distinguish between EGCG solutions (≥10^−3^ mol/L) and DI water based on the diameter of nanogels, which is useful for rough detection of EGCG. Moreover, P(NIPAM-*co*-NMAM) nanogels have a wider temperature range for the simple detection of EGCG.

**Figure 10:**
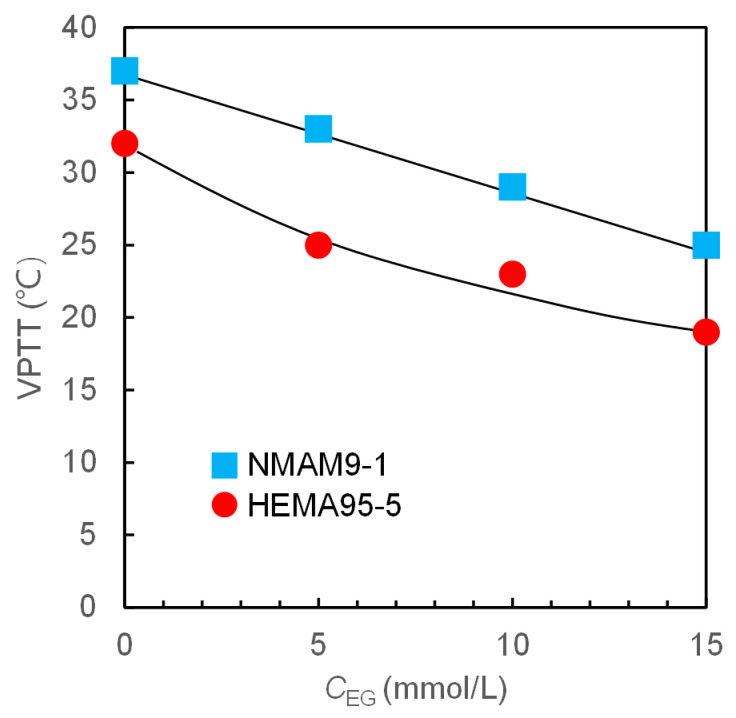
Effect of EG concentration in aqueous solution on the VPTT of P(NIPAM-*co*-NMAM) and P(NIPAM-*co*-HEMA) nanogels. The feeding monomer ratios of NIPAM:HEMA and NIPAM:NMAM are 95:5 and 9:1, respectively.

EG, with fewer phenolic hydroxyl groups, cannot induce the shrinking of nanogels at the tested concentrations (5, 10, and 15 mmol/L) below the VPTT. As the concentration of EG increases in aqueous solutions, the VPTT of nanogels decreases (Figure 10). This means that the temperature at which the nanogels reach a collapsed state decreases with an increase in EG molecules binding in the polymer network. The polymer segments in the hydrogel network can also alter the VPTT of nanogels in EG solutions. When NMAM monomers are introduced into the polymer network, the VPTT of nanogels is higher than those copolymerized with HEMA at the same EG concentration. Therefore, the VPTT can be adjusted not only by the external species in water, such as polyphenols, but also by the copolymerized monomers in the PNIPAM networks.

Interestingly, we found that within a specific temperature range, the diameter of the nanogel is obviously polyphenol concentration dependent. The difference in copolymerized monomers can lead to an increase or decrease in volume of nanogels when exposed to polyphenol with an increased concentration within a specific temperature range. This was not reported in a previous study about pure bulk PNIPAM hydrogel in polyphenol solution [22]. For example, the diameter of P(NIPAM-*co*-HEMA) nanogels increases with the rising concentration of EGCG at temperatures between 37 and 45 °C. In contrast, the diameter of P(NIPAM-*co*-NMAM) nanogels decreases with increasing EG concentration in the temperature range from 29 to 33 °C. The mechanism of nanogels shrinking or swelling with the increase in polyphenol concentration differs. At temperatures from 37 to 45 °C, which are above the VPTT of P(NIPAM-*co*-HEMA) nanogels, water is expelled from the hydrogel network and the nanogels collapse. When EGCG molecules are added to the bulk solution at these temperatures, EGCG molecules diffuse into the shrunken hydrogel network due to their strong hydrophilic and hydrophobic interactions with the polymer chains, leading to an increase in the volume of the nanogels to some extent. Here, EGCG occupies some binding sites of polymer chains that were originally intertwined with other chains, stretching the hydrogel network. However, P(NIPAM-*co*-NMAM) nanogels swell in DI water between 29 and 33 °C, which are below VPTT. The EG molecules in aqueous solutions could disrupt the mechanism of water ordering around the P(NIPAM-*co*-NMAM) polymer chains, leading to the shrinkage of nanogels when equilibrated in EG solution. The results revealed that the introduction of hydrophilic monomers in the PNIPAM network enhances the ability to detect EG molecules below the VPTT, whereas the hydrophobic monomers in the hydrogel network enhance the ability to detect the concentration of EGCG above VPTT, implying different molecular interactions between polyphenols and the nanogel network with different compositions. These nanogels have potential for detecting and loading polyphenols. The results also indicate that the VPTT of thermosensitive nanogels can be adjusted simultaneously by the chemical composition in hydrogel network and polyphenol molecules in solution. 

## 3. Experimental Section

### 3.1. Materials

*N*-isopropylacrylamide (NIPAM, 98%), *N*-hydroxymethyl acrylamide (NMAM, 98%), and 2-hydroxyethyl methacrylate (HEMA, AR) were purchased from Aladdin (Shanghai, China). Potassium persulfate (KPS, AR), *N*,*N*-methylene-bis-acrylamide (BIS, AR), and sodium dodecyl sulfate (SDS, AR), were purchased from Chengdu Kelong Chemicals (Chengdu, China). Ethyl gallate (EG, 98%) and epigallocatechin gallate (EGCG, 98%) were obtained from Macklin (Shanghai, China). Deionized water (18.2 MΩ, 25 °C) was prepared by the Milli-Q Plus water purification system and used in the experiments. 

### 3.2. Preparation of Thermos-Sensitive Nanogels with Different Monomer Ratios

P(NIPAM-*co*-NMAM) and P(NIPAM-*co*-HEMA) nanogels were synthesized with different monomer ratios via the free radical emulsion copolymerization method [30]. Briefly, NIPAM and HEMA with different molar ratios, MBA, and SDS were dissolved together in deionized (DI) water in a 250 mL round-bottom flask with continuous stirring, and the mixture was bubbled with pure N_2_. After a degassing process, the reaction solution was added with KPS, and the polymerization reaction was carried out at 70 °C for 4 h. The obtained nanogel dispersion was purified by dialyzing against DI water using dialysis tubing (MW cutoff 14 KDa) for 7 days with a daily water change. Likewise, P(NIPAM-*co*-NMAM) was obtained by following the same protocol. Nanogels with different compositions were synthesized with the recipes listed in Table 1 and Table 2. Pure PNIPAM microgels were prepared by the precipitation polymerization method [18].

### 3.3. Scanning Electron Microscopy (SEM) 

SEM was conducted using the Thermo Scientific Apreo 2C instrument to examine the morphology of nanogels in the dry state. The aqueous nanogels were diluted and dried on the glass surface and then coated with a thin layer of Au before SEM observing.

### 3.4. Fourier-Transform Infrared (FTIR) Spectroscopy

Nanogels were freeze-dried and the samples were mixed with KBr and pressed to a tablet for the measurement. FTIR spectroscopy (PerkinElmer UATR Two) was conducted on the PNIPAM microgel, P(NIPAM-*co*-NMAM) and P(NIPAM-*co*-HEMA) nanogels within the scanning range of 4000–800 cm^−1^. Background readings were collected and subtracted from each spectrum before the data output.

### 3.5. Thermogravimetric Analysis of Nanogels

Thermal stabilities of nanogels were tested by a thermogravimetric analysis (DTG-60, Shimadzu). The nanogel samples of 4 mg ± 0.3 mg were loaded into aluminum crucibles and heated from 40 °C to 600 °C, with a heating rate of 10 °C/min under a steady nitrogen flow.

### 3.6. DSC

The volume phase transition temperature of nanogels was determined by differential scanning calorimetry (Mettler Toledo, ME-51140313). Hermetically sealed aluminum pans were used in the measurement of thermal properties of nanogels. DSC curves of each sample were obtained from the heating process from 25 to 45 °C at a rate of 0.5 °C/min, under a dry nitrogen rate of 50 mL/min. 

### 3.7. Phase Transition Behaviors of Nanogels at Different Temperatures 

Dynamic light scattering (DLS, Zetasizer Nano-ZS ZEN3600, Malvern, UK) was employed to measure the hydrodynamic diameters and thermo-responsive performance of nanogels within the temperature range of 25 °C to 51 °C, with intervals of 2 °C. The VPTT of the nanogels was determined based on the diameter–temperature curve where the change of nanogel diameter was maximum.

### 3.8. Dynamic Isothermal Volume Changes of Nanogels Induced by EG and EGCG

Investigation of the dynamic volume phase transition behavior of nanogels was carried out by measuring the diameters of nanogels at 25 °C and at certain time intervals. Nanogels were immersed in aqueous solutions with different concentrations of EG and EGCG. Then, the diameter change in the mixed solution was monitored using DLS at certain time intervals. The volume change was defined as:

*V*/*V*_0_ = (*D*/*D*_0_)^3^

where *V* represents the volume of nanogels at different times, *V*_0_ represents the volume of nanogels in aqueous solution, *D* is the diameter of nanogels immersed in polyphenol solution at different time, and *D*_0_ is the diameter of nanogels in aqueous solution at 25 °C before addition of polyphenol.

### 3.9. Thermo-Responsive Phase Transition Behaviors of Nanogels in EG and EGCG Solutions with Different Concentrations

Thermo-responsive phase transition behaviors of nanogels in EG and EGCG aqueous solution were investigated using DLS. Before studying their thermo-responsive phase transition behaviors, the P(NIPAM-*co*-NMAM) and P(NIPAM-*co*-HEMA) nanogels were immersed in EG or EGCG aqueous solution with different concentrations (0, 5, 10, 15 mmol/L respectively) at 5 °C for at least 5 h to ensure that an equilibrium of isothermal diameter change was reached. The temperature of each EG or EGCG aqueous solution containing nanogels was then incrementally increased from 5 to 45 °C at intervals of 4 °C. Each pre-determined temperature was maintained constantly for more than 10 min to ensure the complete equilibrium state of thermo-responsive phase transition.

## 4. Conclusions

In summary, the thermo-responsive nanogels with different monomer compositions were successfully prepared by copolymerizing NIPAM with HEMA or NMAM monomers using emulsion polymerization. The nanogels exhibit a good monodispersity and thermo-responsive phase transition behaviors in water. The VPTT can be adjusted not only by the copolymerized monomers and their amount but also by the polyphenol molecules in the solution environment and their concentrations. The thermosensitive phase transition behavior of nanogels in EG and EGCG aqueous solutions was systematically investigated. The nanogels in EGCG solutions (≥5 mmol/L) are in a shrinking state within 5 to 45 °C and do not show the thermo-responsive phase transition behavior, whereas the nanogels show a thermo-responsive phase transition behavior in EG aqueous solutions, and VPTT shifts to lower temperatures with increasing EG concentration. Interestingly, the diameter of P(NIPAM-*co*-NMAM) nanogels decreases with increasing EG concentration at temperatures between 29 and 33 °C. The diameter of P(NIPAM-*co*-HEMA) nanogels increases with increasing EGCG concentration at temperatures between 37 and 45 °C. The results indicate the different molecular interactions between polyphenols and the hydrogel network with different compositions. The volume phase transition of nanogels could be triggered by the addition of EGCG or EG to a certain critical concentration at a constant temperature below the LCST, providing useful information for developing a simple technology for roughly detecting EG and EGCG concentrations. The results also suggest that the burst-release of drugs from P(NIPAM-*co*-NMAM)-based carriers can be triggered by EGCG molecules (≥10^−3^ mol/L) at physiological temperature [25].

## Figures and Tables

**Table 1:** Recipes for syntheses of P(NIPAM-*co*-NMAM) nanogels.

NIPAM: NMAM	NIPAM (g)	NMAM (g)	MBA (g)	SDS (g)
10:1	1.132	0.1011	0.077	0.025
9:1	1.017	0.1011	0.077	0.025
8:1	0.905	0.1011	0.077	0.025

**Table 2:** Recipes for syntheses of P(NIPAM-*co*-HEMA) nanogels.

NIPAM: HEMA	NIPAM (g)	HEMA (μL)	MBA (g)	SDS (g)
99:1	1.1201	13	0.0314	0.0579
95:5	1.0705	65	0.0310	0.0579
90:10	1.0202	130	0.0311	0.0579
80:20	0.9053	243	0.0311	0.0579

## Data Availability

Data are contained within the article and supplementary materials.

## Supplementary Materials

The following supporting information can be downloaded at: https://www.mdpi.com/article/10.3390/molecules28237823/s1, Figure S1. TGA (a1–d1) and DTG (a2–d2) curves from 25 to 600 °C of the PNIPAM microgels (a) and P(NIPAM-co-NMAM) nanogels with different monomer ratios of 10:1 (b), 9:1 (c), and 8:1 (d). Figure S2. Optical images of EG, nanogels, and nanogels + EG in aqueous solution at 25 °C (a) and 45 °C (b). The PNIPAM-co-HEMA nanogels with monomer ratio of 99:1 (5 mg/mL) were used. The concentration of EG is 10 mmol/L. Table S1 PDI of P(NIPAM-co-HEMA) nanogels in water at 25 °C. Table S2 PDI of P(NIPAM-co-HEMA) nanogels with NIPAM:HEAM = 95:5 in 10 mmol/L EGCG at different temperatures.
