# Supplementary material for: Phase Transition Behaviors of Poly(N-isopropylacrylamide) Nanogels with Different Compositions Induced by (−)-Epigallocatechin-3-gallate and Ethyl Gallate
Source: Molecules. 2023 Nov 28;28(23):7823. doi: 10.3390/molecules28237823 (PMC10708248; doi:10.3390/molecules28237823)
Supplement: Supplementary file 1 [file molecules-28-07823-s001.zip › molecules-2672536-supplementary.pdf]

Supporting Information

Phase Transition Behaviors of Poly(*N*-isopropylacrylamide) Nanogels with Different Compositions Induced by (−)-Epigallocatechin-3-gallate and Ethyl Gallate

Ke Deng [1], Yafei Wang [1], Lei Wang [1], Xianli Fan [1], Zhenyu Wu [1], Xue Wen [2], Wen Xie [2], Hong Wang [2], Zheng Zhou [1], Pengfei Chen [1] and Xianggui Chen [1,*]

[1] School of Food and Bioengineering, Xihua University, Chengdu 610039, China; kelvin_de@126.com (K.D.); wyf@stu.xhu.edu.cn (Y.W.); 212021086000031@stu.xhu.edu.cn (L.W.); 18383013920@139.com (X.F.); zhenyuwu1807@gmail.com (Z.W.); zhouzheng@mail.xhu.edu.cn (Z.Z.); 1220180037@mail.xhu.edu.cn (P.C.)

[2] School of Life Science and Engineering, Southwest Jiaotong University, Chengdu 610031, China; wenxue0703@my.swjtu.edu.cn (X.W.); xiewen@my.swjtu.edu.cn (W.X.)

* Correspondence: chen_xianggui@mail.xhu.edu.cn

Supplementary Figures S1 and S2

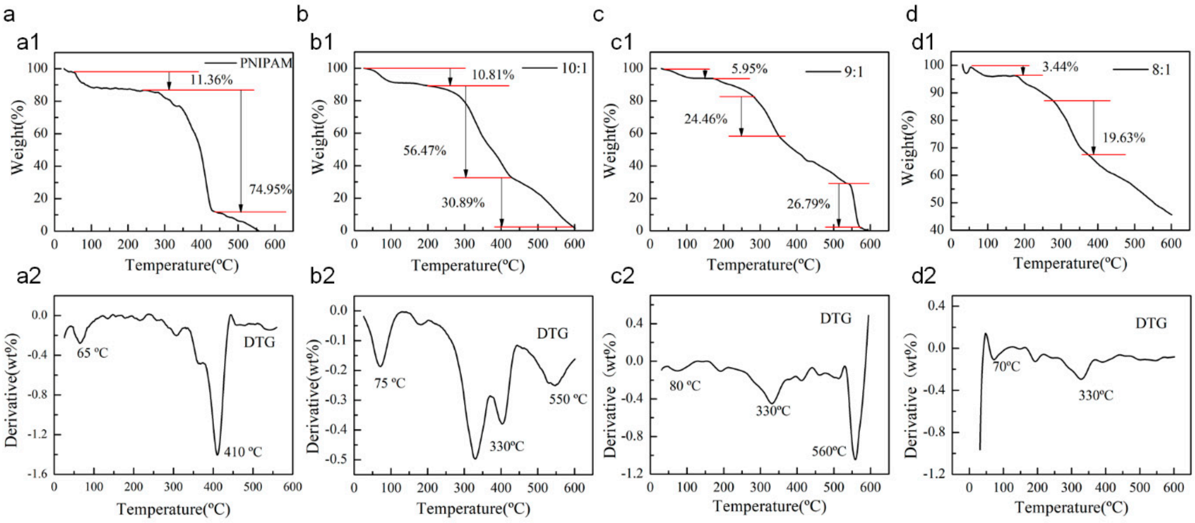

Figure S1. TGA (a1, b1, c1, d1) and DTG (a2, b2, c2, d2) curves from 25 to 600 °C of the PNIPAM microgels (a) and P(NIPAM-*co*-NMAM) nanogels with different monomer ratios of 10:1 (b), 9:1 (c), and 8:1 (d).

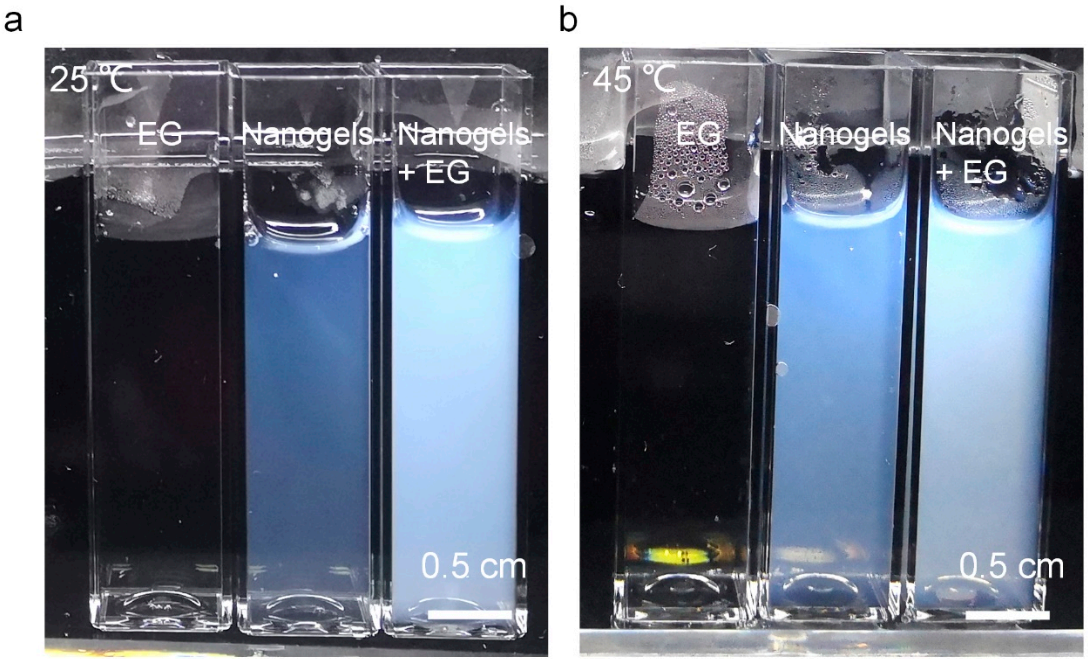

Figure S2. Optical images of EG, nanogels, and nanogels + EG in aqueous solution at 25 ℃ (a) and 45 ℃ (b). The PNIPAM-*co*-HEMA nanogels with monomer ratio of 99:1 (5 mg/mL) were used. The concentration of EG is 10 mmol/L.

Table S1 PDI of P(NIPAM-*co*-HEMA) nanogels in water at 25 °C

Nanogels with different monomer ratios	PDI
NIPAM：HEMA = 99:1	0.066
NIPAM：HEMA = 95:5	0.158
NIPAM：HEMA = 90:10	0.047
NIPAM：HEMA = 80:20	0.087

Table S2 PDI of P(NIPAM-*co*-HEMA) nanogels with NIPAM:HEAM = 95:5 in 10 mmol/L EGCG at different temperatures

Temperatures (°C)	PDI
5	0.110
9	0.107
13	0.117
17	0.096
21	0.109
25	0.1
29	0.11
33	0.089
37	0.108
41	0.095
45	0.102
